# In vitro induction of human embryonal carcinoma differentiation by a crude extract of *Rhazya stricta*

**DOI:** 10.1186/s12906-017-1852-7

**Published:** 2017-06-29

**Authors:** Faisal S. Alagrafi, Abdullah O. Alawad, Nael M. Abutaha, Fahd A. Nasr, Othman A. Alhazzaa, Sultan N. Alharbi, Mohammad N. Alkhrayef, Mohamed Hammad, Ziyad A. Alhamdan, Abdullah D. Alenazi, Mohammad A. Wadaan

**Affiliations:** 1 National Center for StemCell Technology (NCSCT), Life Sciences and Environment Research Institute (LSERI), King Abdulaziz City for Science and Technology (KACST), Riyadh, 11442 Saudi Arabia; 2 Bioproducts Research Chair, Department of Zoology, College of Science, King Saud University, Riyadh, 114570 Saudi Arabia

**Keywords:** Differentiation, NTERA-2 cell lines, Retinoic acid, *Rhazya stricta*, Plant extract

## Abstract

**Background:**

*Rhazya stricta* Decne. is a medicinal plant that is widespread in Saudi Arabia and desert areas of the Arabian Peninsula. Its extract contains alkaloids, tannins, and flavonoids that are involved in different biological activities. The study aim was to evaluate the effects of *Rhazya stricta* plant extracts on the proliferation and differentiation of NTERA-2 (NT2) pluripotent embryonal carcinoma cells.

**Methods:**

Soxhlet extraction was carried out using different solvents to extract stems, leaves and fruit parts of this plant. Cytotoxicity was evaluated by an MTS cell viability assay. The ability of the plant extract to induce cell differentiation was examined phenotypically using an inverted light microscope. The expression of pluripotency markers was investigated by reverse transcriptase polymerase chain reaction (RT-PCR) and immunocytochemistry. Phytochemical screening of chloroform stem extracts was carried out and a chromatographic fingerprint was generated using gas chromatography – mass spectrometry (GC-MS).

**Results:**

Chloroform stem extract induced differentiation of NT2 cells at 5 μg/ml, and the differentiated cells exhibited neurite formation. Following induction of differentiation, there was significant down-regulation of the pluripotency marker genes Oct4 and Sox2. In addition, the surface antigen pluripotency marker, TRA-1-60, was strongly down-regulated. Phytochemical analysis of the extract showed the presence of alkaloids and saponins. The chromatogram revealed the presence of fifteen compounds with different retention times.

**Conclusion:**

Our results demonstrate for the first time that chloroform stem extract of *R. stricta* can induce neuronal differentiation of stem cells at an early stage and may contain potential therapeutic agent that can be used in neurodegenerative diseases.

## Background

Stem cells (SCs) are a type of cells that scattered in the body tissues of multicellular organisms. SCs have the ability to self-renew and differentiate into several types of cells and thus can develop into tissues and organs [1]. The unique features of SCs have been exploited in clinical applications. Mainly in the tissue engineering, cell therapy, and regenerative medicine [2]. The differentiated cells have been used in stem cell research to repair the damaged cells and to evaluate the efficiency and toxicity of new drugs [1].

Medicinal plants are rich in highly biologically active compounds, and at least 25% of the active compounds in current synthetic drugs are isolated from plant sources [3]. It has been estimated that approximately 80% of the world’s population depends on plant-based traditional medicines [4]. Plants contain a high concentration of secondary metabolites such as flavonoids, glycosides, and lignans that involved in different biological activities of plant extracts [5]. Phytochemical screening and extraction are promising for new crude extract or pure compounds that modulate stem cell self-renewal and differentiation. Several bioactive molecules isolated from medicinal plants affect SC proliferation and differentiation. Examples of these molecules include lignin [6], naringin (*Rhizome drynariae*) [7], ginsenosides (*Panax notoginseng*) [8–10], garcinol (*Garcinia indica*) [11], curcumin (*Curcuma longa*) [12–14], and Kuwanon V isolated from the mulberry tree (*Morus bombycis*) [15]

Embryonal carcinoma (EC) stem cells are considered a good model for studying embryonic stem (ES) cell differentiation during embryonic development [16] as they share similar gene expression profiles such as the transcription factors Oct4, Sox2 and Nanog, which are the master regulators of pluripotency and self-renewal [17]. Additionally, the human EC and ES cells express specific embryonic antigens such as SSEA3, SSEA4, TRA-1-60 and TRA-1-81 [16, 18, 19]. TERA2 is one of the oldest extant cell lines that was isolated by Fogh and Trempe [20] from a lung metastasis originating from a testicular germ cell tumor. TERA-2 cells were injected into a nude mouse to form xenograft tumors for clonal sublines. Of these clones, NT2 cells appeared to ‘rescue’ persisting EC cells within the culture [21]. NT2, the best-studied clone that responds in culture to small molecule retinoic acid (RA) by forming postmitotic neurons, was reported for the first time by Andrews [22] and later improved by Pleasure et al. [23] to obtain pure fractions of neuronal cells.

Saudi Arabia contains a diverse array of plant species that are widely used in Saudi’s traditional medicines. [24]. To the best of our knowledge, and based on an internet survey, there is a lack of research showing the effect of Saudi’s plant extracts on stem cell differentiation. Therefore, in the current study, we have investigated the effect of extracts derived from *Rhazya stricta* species on NT2 proliferation and differentiation.

## Methods

### Plant collection and extraction

The *Rhazya stricta* plant was collected between January and February in the spring of 2014 from the Raudhat Al- khafs desert near Riyadh city, Saudi Arabia. The plant was identified and authenticated at the herbarium of the Department of Botany and Microbiology, College of Science, King Saud University, Saudi Arabia. Plants were separated into fruits, stems and leaf parts. First, the plant parts were washed thoroughly with distilled water, air-dried at room temperature and then crushed into powder using an electric blender. The dried powder of each parts (30–50 g) was successively extracted with different polarity of solvents namely n-hexane, chloroform, ethyl acetate, and methanol for 24 h using a Soxhlet apparatus. The crude extracts were centrifuged at 4000 rpm, for 10 min and the supernatants were concentrated to dryness under low pressure at 45 °C in a rotary evaporator. Finally, the crude extracts were dissolved in MeOH, filtered and stored at −80 °C until used. The extraction yield was calculated using the following equation:

$$ Total\  extract ion\  yeild\ \left(\%\right)=\frac{Mass\  of\  the\  extract\ }{Mass\  of\  sample}\times 100 $$

### Cell culture

The NTERA2 cl.D1 [NT2] (ATCC® CRL1973™) human pluripotent embryonal carcinoma cells were cultured in high glucose Dulbeccoʼs Modified Eagle Medium: Nutrient Mixture F-12 (DMEM/F-12, Thermo Fisher Scientific) supplemented with 10% heat-inactivated fetal bovine serum (FBS, Thermo Fisher Scientific), 2 mM L-glutamine, 100 U/ml penicillin and 100 μg/ml streptomycin (Thermo Fisher Scientific) in a humidified incubator (Sanyo, Japan) at 37 °C and 10% CO_2_. The medium was replaced with fresh medium daily. The NT2 cells were passaged every 2–3 days with 0.25% trypsin-EDTA (Thermo Fisher Scientific).

### Assessment of cell viability using the MTS assay

Cell viability was assessed by colorimetric assay using the MTS (3-(4,5-dimethylthiazol-2-yl)-5-(3-carboxymethoxyphenyl)-2-(4-sulfophenyl)-2H–tetrazolium) assay according to the instructions provided by the manufacturer (Promega). Briefly, cells were seeded in 24-well plates (4 × 10^4^/well), allowed 24 h to adhere, treated with different concentrations of plant extract (10, 50, 100, 250, 500, 700, and 1000 μg/ml) and incubated for 48 h in a humidified 10% CO_2_ atmosphere. Methanol (0.01% MeOH) served as the vehicle control. MTS was added at a concentration according to the manufacturer’s instructions for 2 h. The absorbance was measured using a plate reader (BioTek, USA) at a wavelength of 490 nm. The percentage of cell viability after treatment was calculated by assuming 100% viability for the absorbance recorded in the vehicle control. The IC_50_ value was generated from the dose-response curves using Origin 8.5 data analysis and graphing software.

### Induction of the NTERA-2 cell line (NT2) differentiation

The differentiation of SC was induced according to the method of Andrews [22]. The cells were imaged every 2 days using a 1X 50 fluorescence microscope (Olympus, Japan) to observe the cellular differentiation.

### NT2 cell differentiation by retinoic acid (RA) & plant extract

The cells were plated at a density of 15,000 cells/cm^2^ in DMEM/F-12 supplemented with 10% FBS, 2 mM L-glutamine, 100 U/ml penicillin and 100 μg/ml streptomycin. RA-induced cell differentiation was a positive control prepared using 10 μM RA for 3 weeks in a humidified incubator at 37 °C and 10% CO_2_, and the medium was replaced every 2 days. During the RA-induced differentiation process, the NT2 cells were seeded in 3 different T75 flasks, and the time period allowed for differentiation was one week for the 1st flask, two weeks for the 2nd flask, and three weeks for the 3rd flask. Dimethyl sulfoxide (DMSO) was adjusted to 0.001% as a vehicle control for 3 weeks. For extract treatments, NT2 cells were treated with several concentrations of plant extracts (5, 10 and 25 μg/ml) per well of 6-well plates for 2 weeks in a humidified incubator at 37 °C and 10% CO_2_, and the medium was replaced every 2 days. The MeOH (0.001%)-treated cells served as a vehicle control.

### Gene expression analysis by RT-PCR

Total RNA was extracted from NT2 cells treated with chloroform extract of *Rhazya stricta* stems (RS1S CHCL_3_), RA, and vehicle control using Tri-reagent (Sigma) as described by Chomczynski and Mackey [25]. One microgram of RNA was used for cDNA synthesis according to the manufacturer’s instructions (Reverse Transcription System, Promega). PCR amplification reactions were performed in a total volume of 25 μl using GoTaq® Green Master Mix (Promega). The PCR reactions were incubated in the ProFlex PCR system (Applied Biosystems, USA) with the following conditions:94 °C hot start (5 min), denaturation at 94 °C (30 s), annealing temperature of 53–60 °C (30 s; temperature based on the primer), extension at 72 °C (40 s) and post-extension at 72 °C (10 min). The primer sequences and product size are summarized in Table 1. β-actin was used as an internal control, and stem cell markers, including Oct4, Sox2, Nanog, and Klf4, were used to determine the gene expression levels in both undifferentiated and differentiated cells. PCR products were loaded on a 1.2% agarose gel in Tris-acetate-EDTA (TAE) buffer containing SYBR Safe DNA gel stain. The agarose gel was imaged, and the intensity of the gel bands was measured using a Gel Doc XR+ system (Bio Rad, USA). All gene expression was determined for two independent experiments, normalized to β-actin, and the relative levels of stem cell markers after treatment were presented in comparison to the vehicle control.

**Table 1:** List of the primers used in this study

Primer	Sequence	Product size (bp)
h-Oct4	F-CCTCACTTCACTGCACTTGTA R-CAGGTTTTCTTTCCCTAGCT	165
h-Sox2	F- ATGTCCCAGCACTACCAGAG R- GCACCCCTCCCATTTCCC	141
h-Kf4	F- GGTCGGACCACCTCGCCTTACAC R- CTCAGTTGGGAACTTGACCA	172
h-Nanog	F- TTTGTGGGCCTGAAGAAAACT R- AGGGCTGTCCTGAATAAGCAG	116
h-β-actin	F- AAACTGGAACGGTGAAGGTG R- AGAGAAGTGGGGTGGCTTTT	171

### Immunocytochemistry (ICC)

To detect protein expression levels in both undifferentiated and differentiated cells, the monoclonal antibody TRA-1-60 (Santa Cruz Biotechnology) was used as the primary antibody to assess surface antigens on the NT2 cells and the changes that occur upon differentiation induced by RS1S CHCL_3_ extract. Following the treatment, cells were washed and then fixed in 4% paraformaldehyde (20 min) at room temperature. Fixed cells were incubated with blocking buffer (3% FBS in 1× Phosphate buffer saline (PBS)) for 40 min, and then the cells were incubated overnight at 4 °C with TRA-1-60 primary antibody diluted 1:100 in blocking buffer. After incubation, the cells were washed 3 times with 1× PBS and then incubated for 1 h at room temperature on a shaker with FITC-conjugated goat-anti-mouse IgM (Santa Cruz Biotechnology**)** secondary antibody diluted 1:500 in blocking buffer, and the cells were then washed 2 times with 1× PBS. For nuclei staining, the cells were incubated with 1× PBS containing 0.5 μg/ml Hoechst (Sigma) for 5 min. The stained cells were imaged using an In Cell Analyzer 2000 System (GE Healthcare Life Sciences, USA). For the negative control, conditions were kept the same, except that the primary antibody was omitted.

### Phytochemical analysis of plant extract

Phytochemical screening was performed using standard procedures as described by [26–28]. The *Rhazya stricta* extracts were screened for the following phytoconstituents: alkaloids, saponins, flavonoids, and amino acids.

### GC-MS (gas chromatography –mass spectrometry) analysis

Phytochemical investigation of RS1S CHCL_3_ extract was performed on an Agilent 7890A/5975C GC-MS system (Agilent Technologies, USA). The experimental conditions of the GC-MS system were as follows: HP-88 capillary standard column, dimension: 100 Mts, ID: 0.25 mm, film thickness: 0.20 μm. The flow rate of the mobile phase (carrier gas: He) was set at 1.0 ml/min. For the gas chromatography, the temperature program (oven temperature) was 50 °C raised to 250 °C at 5 °C/min, and the injection volume was 2 μl. Samples dissolved in methanol were run fully at a range of 50–650 m/z.

### Statistical analysis

The results are presented as the mean ± standard deviation (± SD) of two independent experiments, and statistical analysis was performed using Student’s t-tests.

## Results

### Polarity and the yield of Soxhlet extraction

**Fig. 1:**
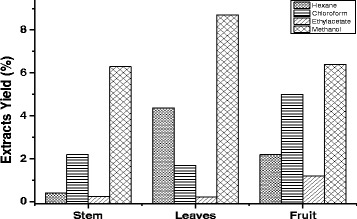
Extract yields of *R. stricta* using different solvents of varying polarity

A total of twelve extracts were prepared from stems, leaves and fruit of *R. stricta*. The yields of extracts using different solvents were obtained in the following order: hexane, chloroform, ethyl acetate and methanol. The extract yields of the plant parts using the various organic solvents are shown in Fig. 1. The highest yields of extraction were obtained from the methanol extraction of leaves (8.7%) and fruits (6.4%), whereas the lowest yields of extraction were from ethyl acetate of leaves (0.23%) and stems (0.25%) (Fig. 1).

### Cytotoxicity of plant extract on NT2 cells

**Fig. 2:**
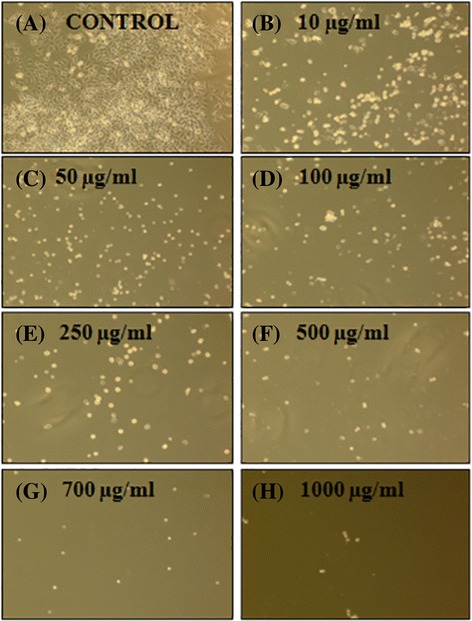
Phase contrast micrographs of RS1S CHCL3 extract-induced changes in cellular morphology. NT2 cells were incubated overnight in 24-well plates in DMEM/F12 medium, and then RS1S CHCL3 extract was added to the medium. After 48 h of treatment, the cells detached from the plates, and the cell shape changed from flat to round in a dose–dependent manner. **a** Vehicle control, **b**–**h** increasing concentrations of the extract were added to the cultures for 48 h. Magnification (10×)

**Fig. 3:**
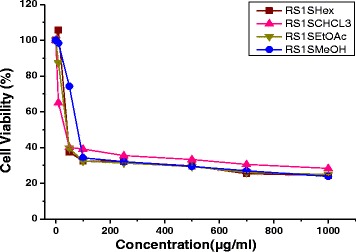
Growth inhibitory activity of solvent extracts of *Rhazya stricta* stems on NT2 cells. The cells were incubated with different concentrations for 48 h, and the cell viability was determined by the MTS assay. The percent inhibition was calculated in comparison to the vehicle control (0.01% MeOH) of two independent experiments. Abbreviations: Hexane extract of *Rhazya stricta* stems (RS1S Hex), chloroform extract of *Rhazya stricta* stems (RS1S CHCL3), ethyl acetate of *Rhazya stricta* stems (RS1S EtOAc), and methanol extract of *Rhazya stricta* stems (RS1S MeOH)

**Fig. 4:**
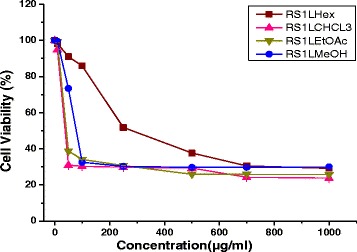
Growth inhibitory activity of solvent extracts of *Rhazya stricta* leaves on NT2 cells. The cells were incubated with different concentrations for 48 h, and the cell viability was determined by the MTS assay. The percent inhibition was calculated in comparison to the vehicle control (0.01% MeOH) of two independent experiments. Abbreviations: Hexane extract of *Rhazya stricta* leaves (RS1L Hex), chloroform extract of *Rhazya stricta* leaves (RS1L CHCL3), ethyl acetate of *Rhazya stricta* leaves (RS1L EtOAc), and methanol extract of *Rhazya stricta* leaves (RS1L MeOH)

**Fig. 5:**
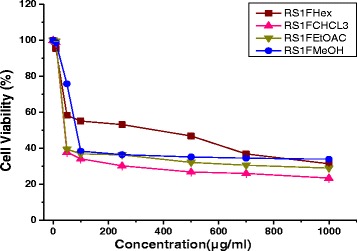
Growth inhibitory activity of solvent extracts of *Rhazya stricta* fruits on NT2 cells. The cells were incubated with different concentrations for 48 h, and the cell viability was determined by the MTS assay. The percent inhibition was calculated in comparison to the vehicle control (0.01% MeOH) of two independent experiments. Abbreviations: Hexane extract of *Rhazya stricta* fruits (RS1F Hex), chloroform extract of *Rhazya stricta* fruits (RS1F CHCL3), ethyl acetate of *Rhazya stricta* fruits (RS1F EtOAc), and methanol extract of *Rhazya stricta* fruits (RS1F MeOH)

MTS cytotoxicity showed that all twelve extracts of *R. stricta* decreased the cell viability in a dose–dependent manner. The chloroform stem extract (RS1S CHCL_3_) showed the most potent inhibitory effects on the proliferation of NT2 cells and altered their morphology dose-dependently. The RS1S CHCL_3_ extract decreased the cells’ attachment resulting in loss of contact with nearby cells. Moreover, the cells’ morphology changed to a rounded shape, and shrinkage was also evident. In contrast, the untreated cells cultured with vehicle control maintained their normal morphology and were adherent to the tissue culture plates (Fig. 2). The IC_**50**_ values for hexane, chloroform, ethyl acetate and the methanolic extract of *R. stricta* stems were 40.7, 33.3, 41.29, and 79.4 μg/mL, *R. stricta* leaves were 278.3, 36.7, 43, and 80, and *R. stricta* fruits were 358, 42.5, 43, and 85.5 respectively (Figs. 3, 4 and 5).

### Induction of NTERA-2 cell line (NT2) differentiation

**Fig. 6:**
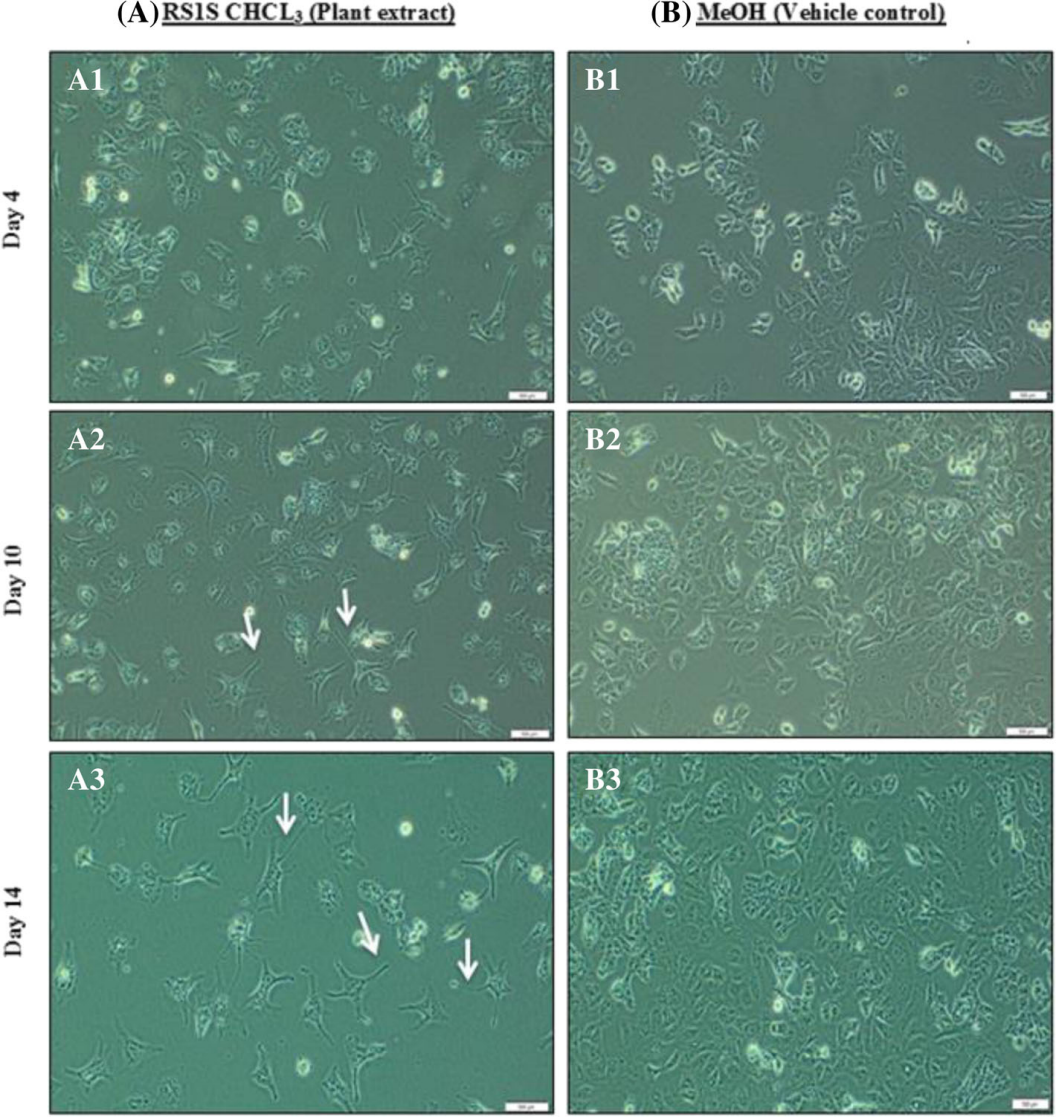
Timeline of NT2 cell differentiation with plant extract. (A1) On day 4, the morphology of NT2 cells with 5 μg/ml of RS1S CHCL3 had begun to change. (A2) On day 10 and (A3) on day 14, the NT2 cells showed short neurites that developed from day 4 until day 14. During incubation, NT2 cells were treated with 5 μg/ml of RS1S CHCL3. (B1, B2 and B3) Vehicle control (0.001% MeOH) cells maintained their morphology over the 14 days. Scale bar, 500 μm. Magnification (10×). Arrows indicate neurite extensions

Among the twelve extracts tested, only the RS1S CHCL_3_ extract at 5 μg/ml induced differentiation of the NT2 cells. The morphology of NT2 cells was characterized by a high nuclear-to-cytoplasmic ratio. During induction of differentiation by RS1S CHCL_3_ extract, a dramatic change in the morphology of NT2 cells was observed starting from the fourth day. The differentiated cells exhibited the neurite formation, but there were some non-differentiated cells that still maintained their morphology (Fig. 6A1). On the tenth day, the cells continued to differentiate and change their morphology with most cells exhibiting neurite formation by day 14 (Fig. 6A2). Hence, after 14 days of treatment, the morphology of the differentiated cells was markedly different compared to that of the 0.001% MeOH vehicle control cells, which maintained their normal morphology (Fig. 6B1, B2 and B3). The differentiated cells showed pronounced reorganization characterized by neurite formation (Fig. 6A3).

**Fig. 7:**
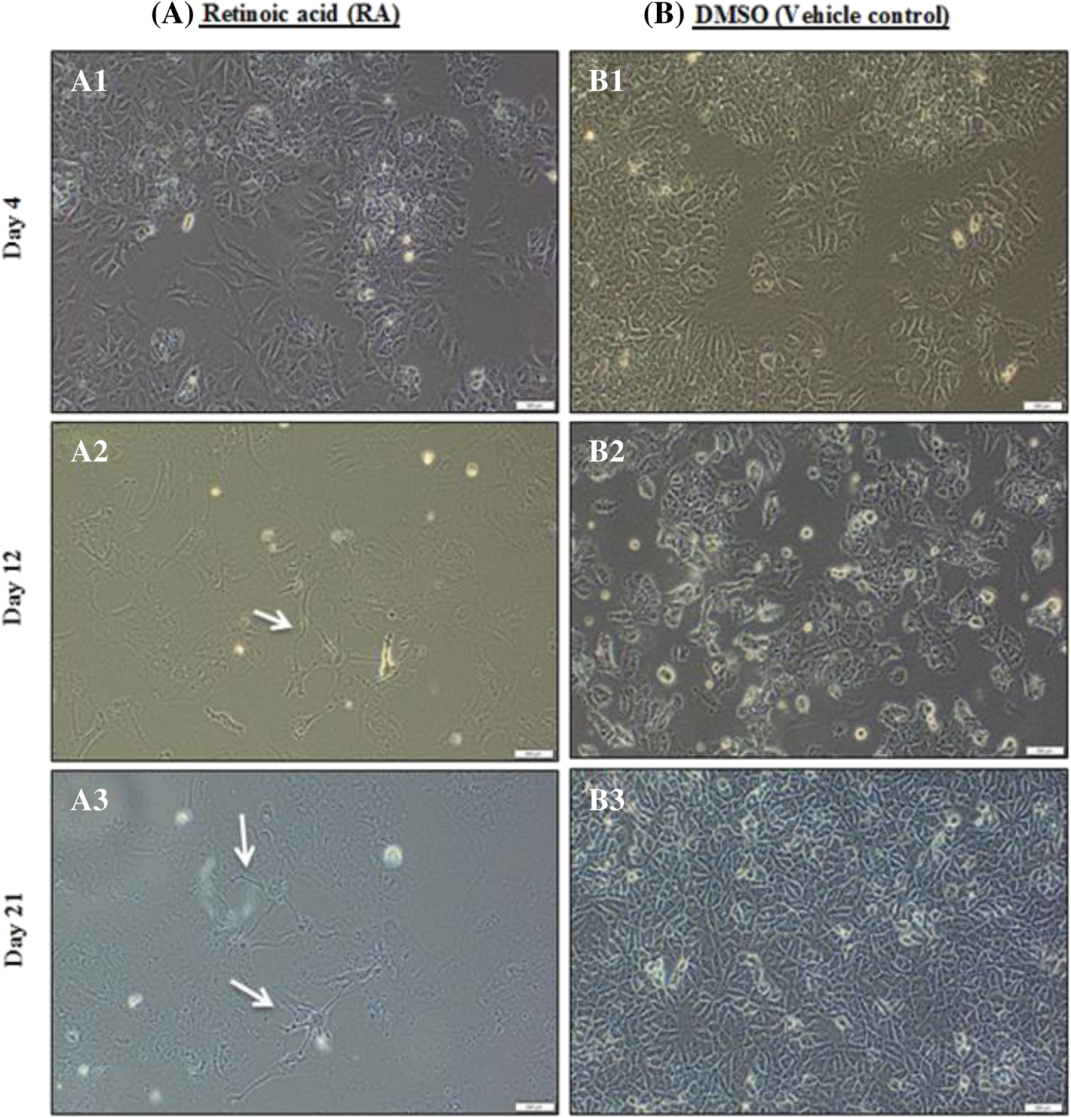
Differentiation of NT2 cells into postmitotic neurons when incubated with 10 μM RA. (A1) On day 4, the morphology of NT2 cells with 10 μM RA had begun to change. (A2) On day 12 and (A3) day 21, the NT2 cells showed neuronal morphology with an extended network over the surface of the culture. (B1, B2 and B3) Vehicle control (0.001% DMSO) cells maintained their morphology over 21 days. Scale bar, 500 μm. Magnification (10×). Arrows indicate neuronal morphology with extended network

The differentiation of NT2 cells into postmitotic neurons induced by 10 μM RA as a positive control was checked at various time points (7 days, 14 days, and 21 days). The differentiation was observed on day 4 when the treated cells changed their morphology (Fig. 7A1). After 1 week, the differentiated cells (neuronal) formed clusters that extended neurites as observed on day 12 (Fig. 7A2). On day 21, the neuronal cells showed neuronal morphology and created a complex cellular network (Fig. 7A3) in contrast to the 0.001% DMSO vehicle control cells, which maintained their normal morphology (Fig. 7B1, B2 and B3).

### Gene expression analysis by RT-PCR

**Fig. 8:**
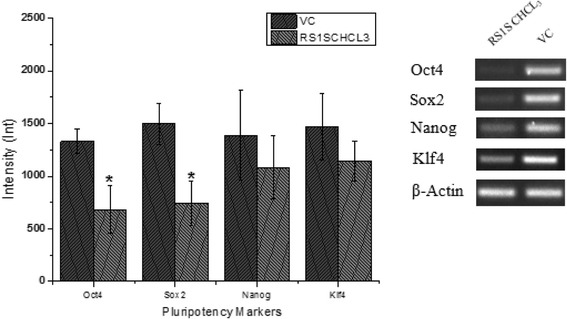
RT-PCR analysis of stem cell genes in NT2 cells after treatment for 14 days with RS1S CHCL3 extract (5 μg/ml). Representative agarose gel electrophoresis patterns of RT-PCR products showed reduced expression of Oct4 and Sox2. All gene expression data were normalized to β-actin as an internal control. Band intensity was measured and the values were expressed as the mean ± SD of two independent experiments. *P < 0.05 compared to vehicle control (VC; 0.001% MeOH)

**Fig. 9:**
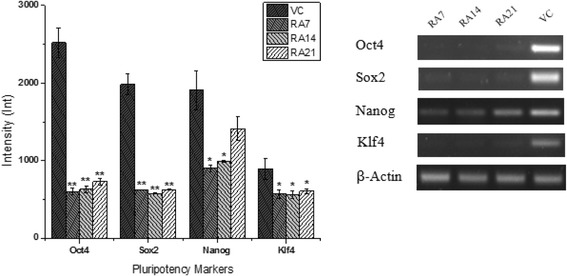
RT-PCR analysis of stem cell genes in NT2 cells at different time points (7, 14, and 21 days) after treatment with retinoic acid (RA). Representative agarose gel electrophoresis patterns of RT-PCR products showed reduced expression of Oct4, Sox2, Nanog, and Klf4. All gene expression data were normalized to β-actin as an internal control. Band intensity was measured and the values were expressed as the mean ± SD of two independent experiments. *P < 0.05, **P < 0.01 compared to vehicle control (VC; 0.001% DMSO)

Gene expression of pluripotency markers Oct4, Nanog, Sox2, and Klf4 was evaluated to assess the morphological changes observed in response to RS1S CHCL_3_ extract and RA treatment. The expression of the stem cell genes Oct4 and Sox2 was significantly downregulated after differentiation with RS1S CHCL_3_ extract at 5 μg/ml for 14 days compared to the vehicle control. In contrast, Sox2 and Oct4 displayed a much lower level of expression compared to Nanog and Klf4 genes after 14 days of treatment as shown in Fig. 8. For further analysis, the cells were exposed to the RA for different amounts of time (7, 14, and 21 days) as a positive control of cell differentiation. On days 7 and 14, the expression of the stem cell genes Oct4, Sox2, Nanog, and Klf4 was significantly downregulated compared to the vehicle control, and this trend continued until day 21, at which point the Nanog gene displayed a much higher level of expression in comparison to days 7 and 14 as shown in Fig. 9.

### Protein expression detection by immunocytochemistry

**Fig. 10:**
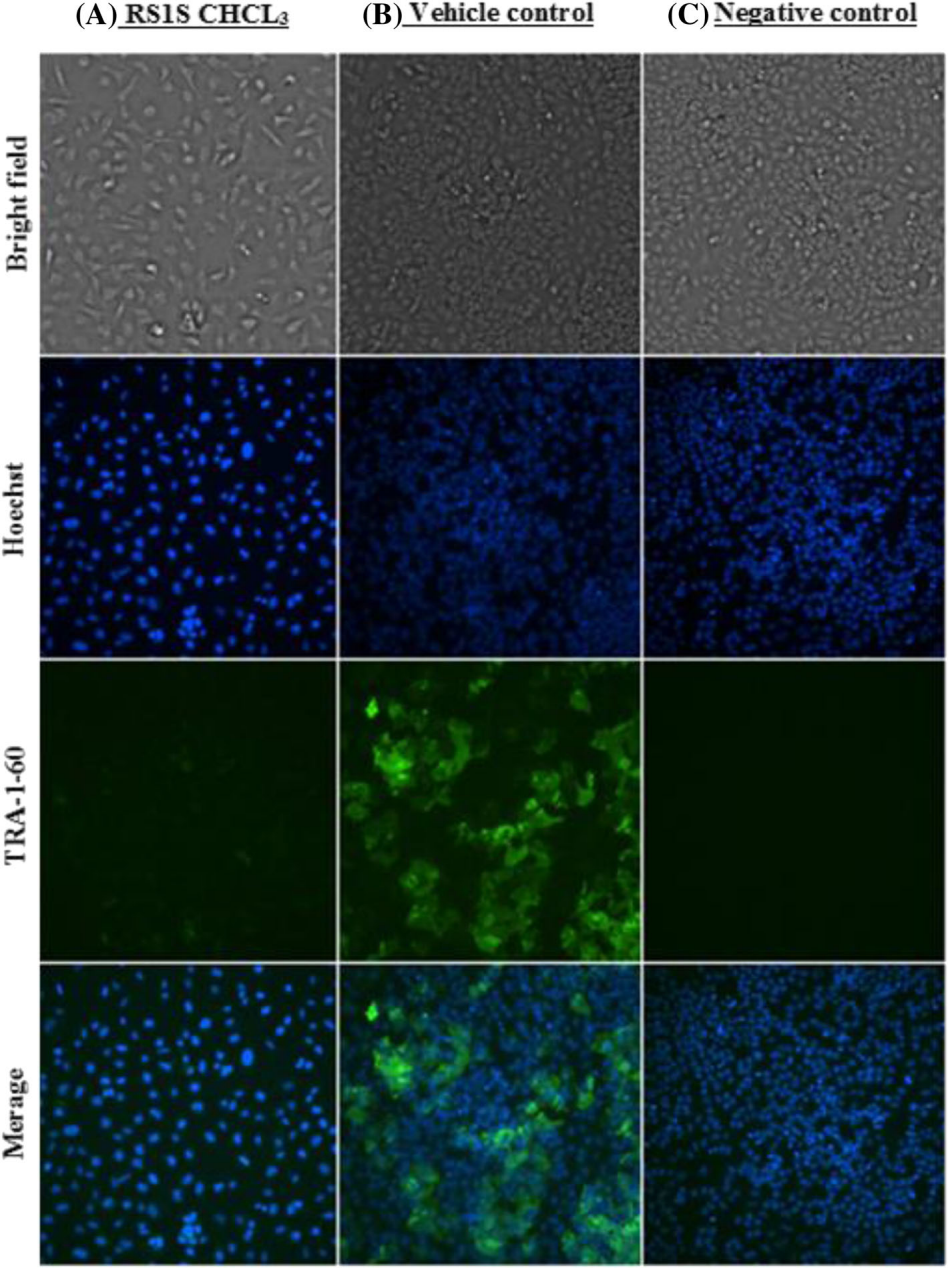
Immunocytochemistry demonstrates the expression of surface antigen (TRA-1-60) in NT2 cells. **a** NT2 cells treated with RS1S CHCL3 extract show downregulation of TRA-1-60 expression. **b** NT2 cells treated with 0.001% MeOH as a vehicle control (VC) show high expression of TRA-1-60. **c** Goat anti-mouse (IgM-FITC) as a negative control. Nuclei stained by Hoechst (blue)

Cell surface antigen expression was analyzed using immunofluorescence to detect the expression of cell surface antigens specific to human EC stem cells. The monoclonal antibody TRA-1-60 is highly expressed in cultures of NT2 cells and it disappears upon cell differentiation. After induction of NT2 cell differentiation with 5 μg/ml of RS1S CHCL_3_ extract for 14 days as shown in Fig. 10, the expression of TRA-1-60 was strongly downregulated in cultures treated with RS1S CHCL_3_ extract compared to the vehicle control. The negative control (without primary antibody) of immunostaining did not show any staining or non-specific binding.

### Phytochemical analysis of RS1S CHCL_3_ extract

The healing properties of medicinal plants are attributed to the presence of various secondary metabolites such as phenols, terpenoids, alkaloids, steroids and others. The phytochemical analysis of RS1S CHCL_3_ extract revealed the presence of active chemical classes of compounds such as alkaloids and saponins, and the absence of phenols, flavonoids, and amino acids (Table 2). The phytochemical analysis of other extracts did not induce the NT2 differentiation are given in Table 2.

**Table 2:** Phytochemical compounds present in *R. stricta* extracts. (+) denotes presence of phytochemical compound, (−) denotes absence of phytochemical compound

Extract	Alkaloids	Saponins	Flavonoids	Phenols	Amino acids
RS1S Hex	**+**	**_**	**_**	**_**	**_**
RS1S CHCL_3_	**+**	**+**	**_**	**_**	**_**
RS1S EtOAc	**+**	**+**	**+**	**+**	**_**
RS1S MeOH	**+**	**_**	**_**	**_**	**_**
RS1L Hex	**_**	**_**	**_**	**_**	**_**
RS1L CHCL_3_	**+**	**+**	**+**	**+**	**_**
RS1L EtOAc	**+**	**+**	**+**	**+**	**_**
RS1L MeOH	**_**	**+**	**+**	**+**	**_**
RS1F Hex	**+**	**_**	**_**	**_**	**_**
RS1F CHCL_3_	**+**	**+**	**_**	**_**	**_**
RS1F EtOAc	**+**	**+**	**+**	**+**	**_**
RS1F MeOH	**+**	**_**	**_**	**_**	**_**

### GC-MS (gas chromatography–mass spectrometry) analysis

**Fig. 11:**
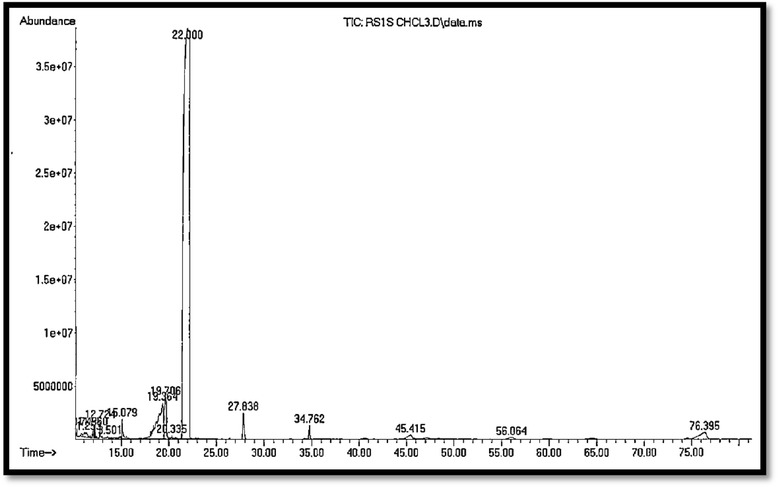
GC-MS chromatogram of chloroform extract of *Rhazya stricta* stems (RS1S CHCL3)

In this study, we describe the potential of GC-MS as a hyphenated technique to generate fingerprints of RS1S CHCL_3_ extract. The GC-MS method was used for the analysis of the volatile compounds. Retention times were used as the main criteria for peak identification using the GC-MS method (Table 3). Fifteen compounds were separated based on different retention times. However, the peak at a retention time of 22 min could indicate a major compound in the crude extract. The chromatogram of RS1S CHCL_3_ extract is shown in Fig. 11.

**Table 3:** Analysis of GC-MS chromatogram of chloroform extract of *Rhazya stricta* stems (RS1S CHCL_3_)

No of Peaks	R.T.min	Peak Height	% of Total
1	10.745	225,624	0.175%
2	11.253	443,723	0.617%
3	11.960	928,609	0.497%
4	12.724	1,422,573	0.367%
5	13.501	172,577	0.154%
6	15.079	1,641,452	1.011%
7	19.364	3,321,601	7.610%
8	19.706	3,841,504	3.288%
9	20.335	296,323	0.086%
10	22.000	38,489,600	81.614%
11	27.838	2,434,016	1.162%
12	34.762	1,227,277	0.496%
13	45.415	368,104	0.697%
14	56.064	174,135	0.333%
15	76.395	9,645,696	1.894%

## Discussion

The effects of 12 plant extracts obtained from different parts of *Rhazya stricta* on the proliferation and differentiation of NT2 cells were investigated. Parts of *R. stricta* (leaves and flowers) are used in traditional medicine for the treatment of rheumatism and allergy [29]**,** and it has also been reported to have antioxidant activity in rats [30]. Additionally, the crude alkaloid extract of *R. stricta* has been found to induce apoptosis in human lung cancer cells [31]. Until now, no studies have shown the induction of SC differentiation by *R. stricta* extract. Therefore, this study is the first to demonstrate the induction of SC differentiation by *R. stricta* extracts. It was found that only RS1S CHCL_3_ induced the differentiation of the NT2 cell line at a concentration of 5 μg/ml. NT2 cells are a pluripotent human embryonal carcinoma cell line, which may differentiate into many different cell types if exposed to certain stimuli [22, 32, 33].

The morphology of NT2 cells is characterized by little cytoplasm, prominent nucleoli and growth in the form of clusters [34, 35]. In the presence of RA, NT2 cells undergo neuronal differentiation and rapidly lose their morphology to exhibit a single axon with multiple dendrites, which is a typical neuronal morphology [23]. Moreover, gene expression profiles were assessed for the NT2 cells exposed to RA for 2–4 weeks. In the first phase of differentiation, on day 3, there was high expression of hat1, which remained constant, and rapid accumulation of nestin, suggesting a marker of neuroprogenitors, although it then decreased dramatically. In the second phase, neurod1 exhibited expression on the 7th–14th days of differentiation, which indicates the neuroprogenitors exited the cell cycle. There was enhanced neural differentiation and the synaptophysin showed expression on the 3rd day and gradually increased until the third phase of differentiation [36]. The RS1S CHCl_3_ extract changed the NT2 cell morphology during the 14 days of the treatment. The differentiated cells exhibited the neurite formation (cells with more than 3 neurites). In accordance with these reports and the characterization of neuronal morphology, the differentiated cells tended to be differentiated into a neuronal lineage.

Cell viability was monitored by MTS assay to distinguish a cytotoxic response from an induction of differentiation. The toxicity of RS1S CHCL_3_ extract started approximately 10 μg/mL after 2 days of treatment with IC_**50 =**_ 33.3, while the high concentrations were highly cytotoxic. However, the proliferation of NT2 cells typically decreases during cellular differentiation, and the RS1S CHCL_3_ extract at a concentration of 5 μg/ml over 14 days of treatment affected cell proliferation.

The expression of transcription factors, including Oct4, Sox2, and Nanog, in pluripotent cells is important in self-renewal and differentiation [37]. These factors, along with other transcription factors such as Klf4 and c-myc, can be used to reprogram somatic cells to become induced pluripotent stem cells (iPSCs) [38]. The activation of pluripotency markers was found to effectively maintain SC in the undifferentiated state. In contrast, if the expression of pluripotency markers is downregulated, it will initiate differentiation [37]. Based on these reports, our results are in accordance with the published literature, as the treated cells had significantly downregulated transcript levels of Oct4 and Sox2 compared to the vehicle control. However, Nanog and Klf4 were also downregulated, although not significantly, after 14 days of treatment by RS1S CHCL_3_. RA also showed significantly downregulated transcript levels of stem cell genes, including Oct4, Nanog, Sox2, andKlf4, on the 7th -14th day of differentiation. The downregulation continued until the 21st day of differentiation except for the Nanog gene, which showed a higher expression at day 21 compared to day 7 and day 14, although this could be due to different stages of differentiation. In previous studies, Reynertson found that ethyl acetate fractions of *Suriana maritime* leaf and stem extracts downregulated Oct4, Nanog and Rex1 genes at 18, 48, and 96 h of treatment and induced differentiation [39]. Lignin suppressed the undifferentiation markers Nanog and Rex1 and promoted the expression of the neuroectodermal markers sox1 and Otx2 that differentiate mouse ES cells into neuroectodermal cells [6].

Various studies have reported that NT2 stem cells are characterized by expression of the surface antigen TRA-1-60 in the undifferentiated state [40, 41]. When the NT2 cells are induced to differentiate into neurons by RA, the differentiation was marked by downregulation of this surface antigen as described by Andrews [22]. This loss of TRA-1-60 expression is accompanied by the acquisition of another antigen such as A2B5, a marker for neural differentiation [42]. TRA-1-60 protein expression was checked by immunocytochemistry to confirm the differentiation. Upon exposure to RS1S CHCL_3_ extract, treated cells showed downregulation of the surface antigen TRA-1-60 after 14 days of treatment in comparison to the vehicle control where TRA-1-60 was highly expressed, which indicates that the cells are in the undifferentiated state.

Phytochemical substances isolated from plants have untold biological and therapeutic potential. They are a rich source for drug discovery, food supplements, and nutraceuticals [43]. Many alkaloids and a few flavonoids have been structurally elucidated from *R. stricta* (mostly from the leaves but also from other parts of the plant) found in Saudi Arabia, Pakistan, India and United Arab Emirates [44]. Some of the alkaloids isolated from *R. stricta* are antirhine, geissoschizine [45], 16-e-Z-isositsirikine, vallesiachotamine, sewarine, tetrahydrosecamine, polyneuridine [46, 47], aspidospermiose, strictibine, 1-carboxymethoxy-β-carboline [48], and stemmadenine [49]. The preliminary phytochemical tests provide insight into the isolation and characterization of active compounds leading to drug discovery and development. Our findings showed the presence of alkaloids and saponins, and the absence of phenols, flavonoids, and amino acids in RS1S CHCL_3_ extract. Plant collection may occur in different seasons, during different stages of plant development, or in the presence of different environmental factors. Due to these factors, the active compound/s may not be present. Additionally, the freezing and thawing cycle of the extract, oxidation, degradation, hydrolysis, thermal instability, and photodegradation may result in the loss of the activity of the compound/s or changes in the solubility of the extract [50]. Therefore, the best method to overcome this problem is to generate a fingerprint of the extracts or fractions to monitor the production and stability of the extract over time and similarity of the extract when recollection occurs. Such quality control is a key issue in herbal medicine development. The fingerprint technique has been widely established as a useful method for the quality control and evaluation of herbal extracts [51, 52]. Several methods are used for fingerprinting of natural products such as HPLC, IR, HPTLC and GC-MS. Lu et al., [53] used GC-MS to create a fingerprint of *Houttuynia cordata*. They found 15 compounds that could be used as markers to identify and evaluate the consistency in 40 different factories and different batches. Paul et al. [54] analyzed volatile substances in *Meum athamanticum* to generate a profile of 46 components that was used to monitor seasonal and geographic chemical variation. In this study, we described the fingerprints of RS1S extract that could allow for monitoring the stability and comparing the composition of selected extracts for further isolation and characterization of the active principle/s as well as other in vivo and in vitro studies in the future.

## Conclusions

We have shown for the first time that the chloroform extract of *R. stricta* stems (RS1S CHCL_3_) from Saudi’s medicinal plants induces cell differentiation in pluripotent embryonal carcinoma cell lines (NT2, in vitro model). All the pluripotency markers, Sox2, Oct4, Nanog, Klf4, TRA-1-60, were downregulated after 14 days of treatment. Our present study demonstrates the presence of alkaloids and saponins in this extract, which could be responsible for the cell differentiation.

Our results showed the differentiated cells tended to be neuronal cells according to their morphology with short neurite formations. This study suggests that RS1S CHCL_3_ extract contains bioactive compounds that play roles in neuronal differentiation at the early stage. Hence, this extract may be effective as a therapeutic agent in neurodegenerative diseases.

## Abbreviations

DMEM/F12: Dulbeccoʼs Modified Eagle Medium: Nutrient Mixture F-12
DMSO: Dimethyl sulfoxide
EC: Embryonal carcinoma
ES: Embryonic stem
FBS: Fetal bovine serum
GC-MS: Gas chromatography – mass spectrometry
IC_50_: Half maximal inhibitory concentration
ICC: Immunocytochemistry
Methanol: MeOH
MTS: (3-(4,5-dimethylthiazol-2-yl)-5-(3-carboxymethoxyphenyl)-2-(4-sulfophenyl)-2H–tetrazolium)
NT2: NTERA-2 cell line
PBS: Phosphate buffer saline
RA: Retinoic acid
RS1F CHCL_3_: Chloroform extract of *Rhazya stricta* fruits
RS1F EtOAc: Ethyl acetate of *Rhazya stricta* fruitss
RS1F Hex: Hexane extract of *Rhazya stricta* fruits
RS1F MeOH fruits: Methanol extract of *Rhazya stricta* stems
RS1L CHCL_3_: Chloroform extract of *Rhazya stricta* leaves
RS1L EtOAc: Ethyl acetate of *Rhazya stricta* leaves
RS1L Hex: Hexane extract of *Rhazya stricta* leaves
RS1L MeOH stems: Methanol extract of *Rhazya stricta* leaves
RS1S CHCL_3_: Chloroform extract of *Rhazya stricta* stems
RS1S EtOAc: Ethyl acetate of *Rhazya stricta* stems
RS1S Hex: Hexane extract of *Rhazya stricta* stems
RS1S MeOH stems: Methanol extract of *Rhazya stricta* stems
RT-PCR: Reverse transcriptase polymerase chain reaction
SC: Stem cell
SD: Standard deviation
TAE: Tris-acetate-EDTA
VC: Vehicle control
